# Sex differences in nutrient gaps among active adults

**DOI:** 10.1017/jns.2025.10070

**Published:** 2026-01-02

**Authors:** Grant M. Tinsley, Marleigh Hefner, Philip Sapp, Jeremy Townsend, Christian Rodriguez, Christine Florez

**Affiliations:** 1 Department of Kinesiology & Sport Management, Texas Tech University, Lubbock, TX, USA; 2 Department of Nutritional Sciences, Texas Tech University, Lubbock, TX, USA; 3 Research, Nutrition, and Innovation, AG1, Carson City, NV, USA; 4 HealthHuman Performance, Concordia University Chicago, Health & Human Performance, River Forest, IL, USA; 5 Pennington Biomedical Research Center, LSU System, Baton Rouge, LA, USA

**Keywords:** Exercise, Micronutrients, Minerals, Vitamin C, Vitamin D, Vitamins

## Abstract

Nutrient gaps are differences between recommended and actual intakes and are often based on the estimated average requirement (EAR), the average daily intake estimated to meet the requirement of 50% of healthy individuals. While nutrient gaps have been established in the general population, their presence in exercising adults has not been extensively investigated. In the present analyses, 681 dietary recalls were obtained from 226 healthy exercising adults (154 F, 72 M) using the Automated Self-Administered 24-h (ASA24®) Dietary Assessment Tool. Intakes of seventeen vitamins and minerals were compared to corresponding EAR values to determine if nutrient gaps were present. Additionally, the potential for sex differences in absolute and relative nutrient intakes was examined. Median intakes of vitamin D fell below the EAR in both female and male adults, with the median intake of vitamin E below the EAR in female adults only (*p* ≤ 0.003 for each). In female participants, >50% exhibited intakes below the EAR for calcium, folate, magnesium, vitamin A, vitamin C, vitamin D, and vitamin E. In male participants, >50% exhibited intakes below the EAR for vitamin C, vitamin D, and vitamin E. Sex differences were present for intakes in sixteen of seventeen micronutrients (*p* < 0.001 for each), with lower intakes observed in female adults. Collectively, the present analyses indicate underconsumption of some micronutrients, particularly in exercising female adults. The potential to improve vitamin and mineral intakes and attendant health and performance outcomes through targeted interventions in exercising adults should be explored in future research.

## Introduction

A comprehensive global analysis recently indicated that the majority of the world population consumes inadequate quantities of micronutrients from foods.^(1)^ Over 5 billion people were deemed to have insufficient intakes of iodine, vitamin E, and calcium; while over 4 billion underconsumed iron, riboflavin, folate, and vitamin C. Additionally, sex differences were observed in these nutrient gaps, with more pronounced inadequate intakes of iodine, vitamin B12, iron, selenium, calcium, riboflavin, and folate in female adults and relatively poorer intakes of magnesium, vitamin B6, zinc, vitamin C, vitamin A, thiamine, and niacin in male adults.^(1)^ The analysis producing these results was conducted using the Global Dietary Database, which includes 185 countries and ∼7.5 billion people. As such, the results provide a valuable indication of global micronutrient intake trends. However, detailed examination within specific countries and populations is still warranted to provide a more nuanced view of micronutrient intakes.

Micronutrients serve as essential cofactors that support energy metabolism and a robust immune and antioxidant defense system.^(2)^ Over time, insufficient micronutrient intake can contribute to a state of deficiency, adversely affecting quality of life, performance, and health-related outcomes (e.g. anemia, fractures, acute infection risk) across sexes.^(3–6)^ This relationship is likely bidirectional, evidenced by observational and experimental literature linking optimal intake of micronutrients (e.g. vitamins A, C, D, and zinc) with reduced risk of acute infection, such as viral respiratory infections.^(2)^ Broadly, a ‘nutrient gap’ refers to the difference between recommended and actual intakes of a particular nutrient. While nutrient gaps can be calculated relative to various nutrient intake reference values, the estimated average requirement (EAR) is often used.^(7–11)^ The EAR for a nutrient, established by the Food and Nutrition Board of the Institute of Medicine in the United States, indicates the average daily intake level estimated to meet the requirement of 50% of healthy individuals. The percentages of individuals whose nutrient intakes fall below the EAR values have been presented based on results from the national food survey *What We Eat in America*, conducted by the United States Department of Agriculture in conjunction with the US Department of Health and Human Services.^(8–11)^ These data indicate that the micronutrients most frequently consumed in quantities below the EAR are vitamin D, vitamin E, magnesium, vitamin A, and calcium, for which less than half of the population is estimated to consume more than the EAR when collapsed across race and ethnicity categories (Table 1). Additionally, possible differences in micronutrient intakes based on sex and race or ethnicity are observed for some but not all nutrients.

**Table 1.** Percentage of US adults consuming less than the estimated average requirement. Data from food and beverages in adults aged 19 + are shown. Data obtained from Usual Nutrient Intake tables, What We Eat in America, NHANES 2017–2020 ^(8–11)^

	Non-Hispanic white		Non-Hispanic black		Non-Hispanic Asian		Hispanic	
Nutrient	M	F	M	F	M	F	M	F
Calcium	26	59	47	72	48	70	29	52
Copper	5	13	14	24	<3	6	5	14
Folate	9	27	18	35	5	12	7	26
Iron	<3	10	<3	15	<3	11	13	<3
Magnesium	55	50	77	66	52	41	54	48
Niacin	<3	<3	<3	<3	<3	4	<3	<3
Phosphorus	<3	<3	<3	<3	<3	<3	<3	<3
Riboflavin	4	<3	15	11	12	6	7	5
Selenium	<3	<3	<3	<3	<3	<3	<3	<3
Thiamine	3	12	10	18	3	7	4	12
Vitamin A	48	37	69	56	59	42	61	43
Vitamin B12	4	10	9	14	12	16	5	10
Vitamin B6	<3	<3	<3	<3	<3	<3	7	15
Vitamin C	55	50	58	45	43	25	50	36
Vitamin D	94	>97	96	>97	93	>97	94	>97
Vitamin E	68	84	80	86	77	87	76	86
Zinc	20	18	39	31	28	21	17	17

M, male; F, female.

Collectively, nutritional surveys indicate nutrient gaps at the global and national level. However, the presence of such nutrient gaps in specific subpopulations is less clearly established. One such group is exercising adults; it has been noted that exercise stresses numerous metabolic pathways that require micronutrients, and chronic exercise training leads to adaptations that could increase the need for micronutrients.^(12)^ Additionally, some active individuals intentionally restrict their dietary intake as a method of weight or body composition control and sometimes avoid specific food groups, which could increase the risk of inadequate micronutrient intakes.^(12)^ While some data indicate potential sex differences in these practices,^(13)^ additional investigation is needed to better clarify this occurrence. Based on these considerations, the purpose of the present analyses was to evaluate the presence of nutrient gaps within a healthy, exercising population and to determine if sex differences in nutrient gaps are present.

## Methods

### Participants

Data from five previous studies of active adults were pooled for the present post-hoc analyses. University institutional review board approval was obtained prior to each of these studies (IRB2023-1045, IRB2023-561, IRB2022-933, IRB2021-649, IRB2019-356). Written informed consent was obtained from each participant within these studies, and each study was conducted in accordance with the ethical principles of the Declaration of Helsinki. All studies were conducted in Lubbock, Texas, USA (33.6° N, 101.9° W), between 2019 and 2024, with participant assessments occurring in January (7.7%), February (14.5%), March (17.9%), April (14.2%), May (5.4%), September (7.1%), October (14.5%), November (16.5%), and December (2.3%). Each study involved generally healthy, active, non-pregnant participants, ranging from recreationally active to those engaging in frequent, structured exercise training. In several of the studies, an exercise history questionnaire was included, allowing for a description of the exercise training history and current exercise participation of most study participants (Table 2). This custom questionnaire elicited information about participants’ training history (e.g. years trained) and typical weekly frequency of any exercise participation, resistance training participation, and endurance training participation.

**Table 2.** Participant characteristics

	All (*n* = 226)		F (*n* = 154)		M (*n* = 72)	
Variable	Mean	SD	Mean	SD	Mean	SD
Age (y)	22.5	3.7	22.2	3.6	23.1	3.8
Height (cm)	167.5	8.9	163.4	6.4	176.4	6.7
Body mass (kg)	69.7	12.9	64.9	10.6	80.0	11.4
BMI (kg/m^2^)	24.7	3.5	24.3	3.4	25.7	3.5
Body Fat (%)	27.9	8.4	32.0	6.3	19.1	5.0
FFMI (kg/m^2^)	17.7	2.9	16.3	1.8	20.8	2.4
Energy intake (kcal/d)	2141	980	1748	579	2982	1128
Years traineda	5.9	4.8	5.6	4.9	7.5	4.6
Exercise frequency (d/week)^a^	3.5	1.6	3.3	1.6	4.7	1.3
RT frequency (d/week)^a^	2.0	1.8	1.7	1.7	3.6	1.7
ET frequency (d/week)^a^	1.7	1.4	1.8	1.5	1.5	1.2

M, male; F, female; SD, standard deviation; FFMI, fat-free mass index; RT, resistance training; ET, endurance training.

^a^Sample sizes for exercise history questionnaire were *n* = 162 (all), *n* = 136 (F), and *n* = 26 (M).

### Nutritional intake assessment

To assess nutritional intake, participants completed the Automated Self-Administered 24-h (ASA24®) Dietary Assessment Tool, a free web-based tool from the National Institutes of Health that enables self-administered 24-h diet recalls.^(14)^ The ASA24® tool asks users to provide information about the intakes of both foods and supplements, and participants were asked to complete the assessment in its entirety. The total nutrient intakes, reflective of both foods and supplements entered by participants, were used in the present analyses. For each study, the current version of ASA24® at the time of data collection was used (including ASA24-2018, ASA24-2020, and ASA24-2022), with multi-pass questions and portion size images enabled. Intakes of vitamins and minerals included in the ASA24® assessment were compared to the corresponding EAR values.^(15)^ All micronutrients that have an established EAR value and that are also included in ASA24® output were included in the analyses. Accordingly, ten vitamins (folate, niacin, riboflavin, thiamine, vitamin A, vitamin B12, vitamin B6, vitamin C, vitamin D, and vitamin E) and seven minerals (calcium, copper, iron, magnesium, phosphorus, selenium, and zinc) were included in the present analyses. Across all studies, participants received standardized in-person instructions for the completion of the ASA24® assessment within a single research laboratory. Due to day-to-day variability in nutritional intake, nutrient values were averaged across dietary records for each individual participant.

### Statistical analyses

Sample size was determined by the availability of previously collected data rather than formal statistical procedures. One-sample Wilcoxon signed-rank tests were performed to determine if the nutrient intakes, expressed nutrient gap scores (i.e. percentage of the EAR for the corresponding nutrient), differed from 100% (i.e. the EAR for each nutrient). Wilcoxon rank-sum tests, also known as Mann-Whitney U tests, were performed to determine if differences in absolute (raw units) or relative (nutrient gap scores expressed as percentages) intakes were present between sexes. The percentage of individual nutrient gap scores <100% (i.e. intakes below the EAR) was also calculated for each nutrient. To account for separate statistical tests being performed for seventeen nutrients, statistical significance was accepted at a Bonferroni-corrected level of *p* < 0.003 (i.e. 0.05/17). Sensitivity analysis was performed after exclusion of individuals with potentially misreported data. Specifically, the method described by Bajunaid et al.^(16)^ was used to identify individuals who had potentially misreported energy intake. These individuals were excluded from the sensitivity analysis, leaving only individuals who were deemed to have plausible energy intakes via this method. The sensitivity analysis results are presented in Supplementary Tables 1–2 and Supplementary Figures 1–3. Data analyses were performed in R (v. 4.4.2).

**Figure 1.**
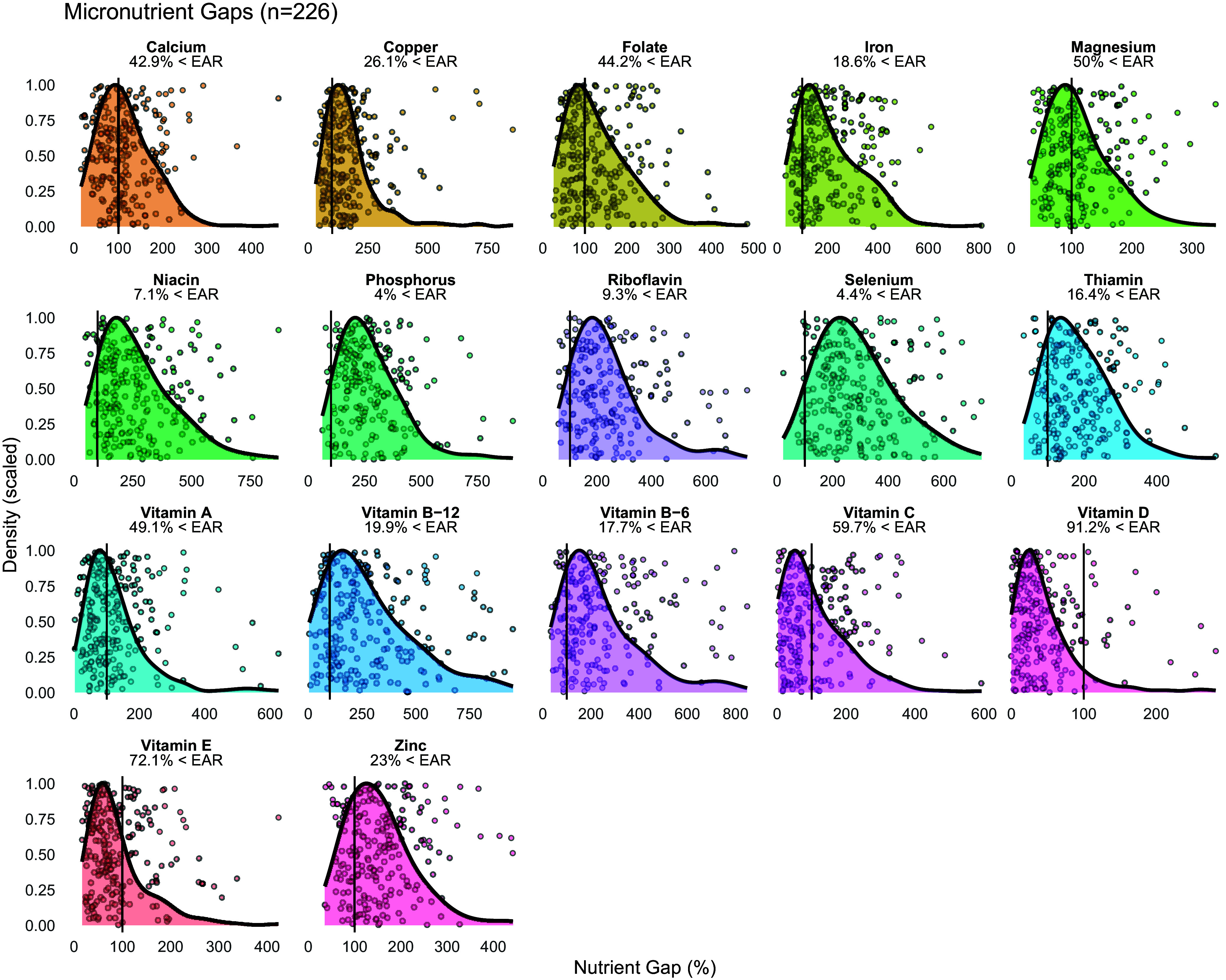
Micronutrient gaps in all participants. Data from 226 male and female participants are displayed. Density plots indicate the distribution of nutrient intakes, expressed as nutrient gap percentages (i.e. percentages of the Estimated Average Requirement [EAR], with a score of 100% indicating intake at the level of the EAR). Vertical black lines in each panel indicate the EAR (i.e. score of 100%) for each nutrient, and the percentage of participants whose intakes were below the EAR is indicated. Values beyond 1000% are not displayed for the purposes of visualization.

## Results

### Participants and ASA24® records

A total of 226 participants and 681 individual ASA24® records were included in the primary analyses, comprised of 301 records from 154 female participants and 380 records from 72 male participants. 47.3% of participants had one record, 23.9% had two records, 18.5% had three to four records, and 10.3% had more than four records. For the 162 participants with completed exercise history questionnaires, the mean ± SD years of exercise training was 5.9 ± 4.8 years, with a current exercise frequency of 3.5 ± 1.6 days per week (Table 2). From study records, it was estimated that ∼81.5% of ASA24® records represented weekday intakes while ∼18.5% of records represented weekend intakes. The sensitivity analysis included data from 159 participants (98 female and 61 male; Supplementary Table 1).

### Vitamin Intakes

**Figure 2.**
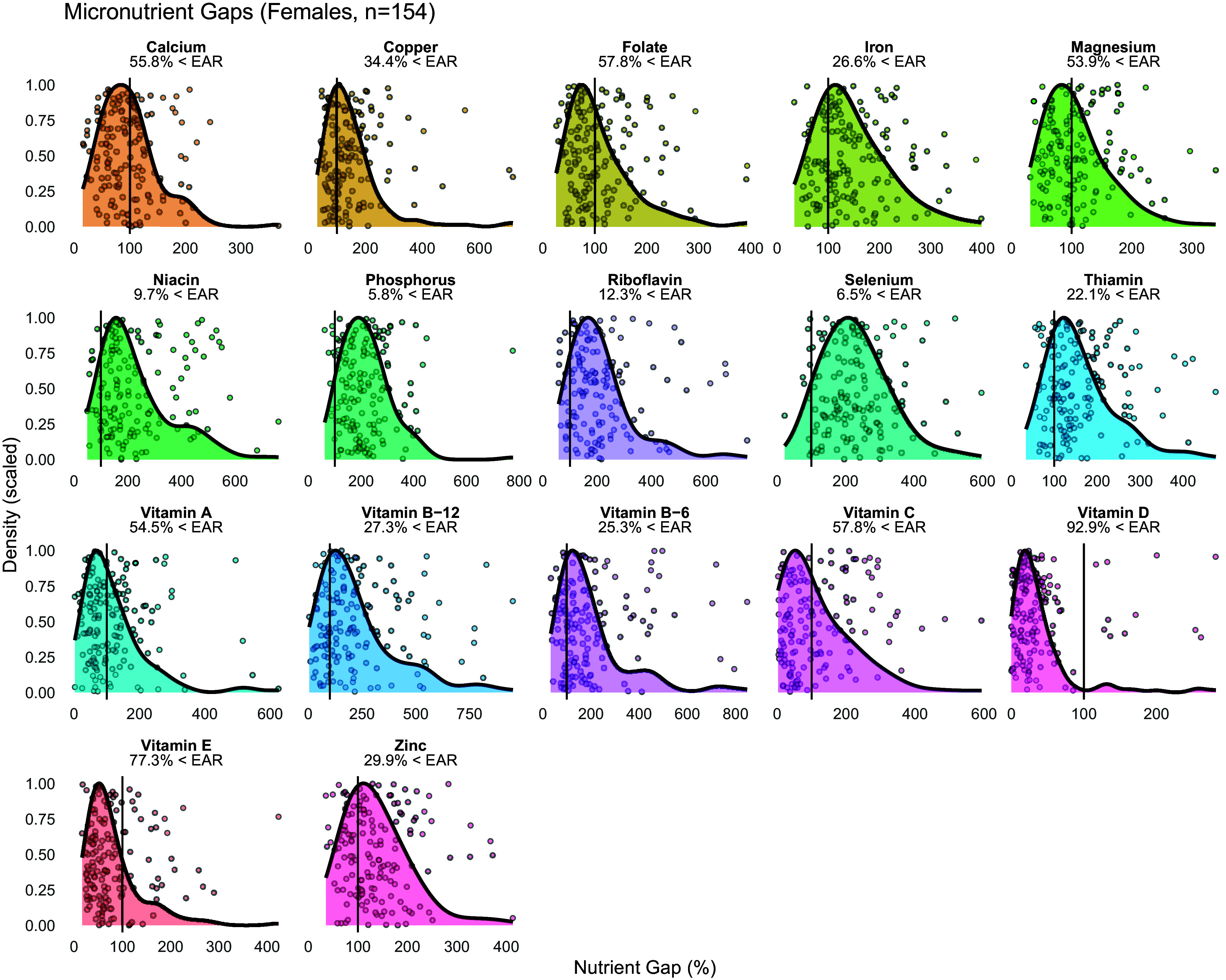
Micronutrient gaps in female participants. Data from 154 female participants are displayed. Density plots indicate the distribution of nutrient intakes, expressed as nutrient gap percentages (i.e. percentages of the Estimated Average Requirement [EAR], with a score of 100% indicating intake at the level of the EAR). Vertical black lines in each panel indicate the EAR (i.e. score of 100%) for each nutrient, and the percentage of participants whose intakes were below the EAR is indicated. Values beyond 1000% are not displayed for the purposes of visualization.

**Figure 3.**
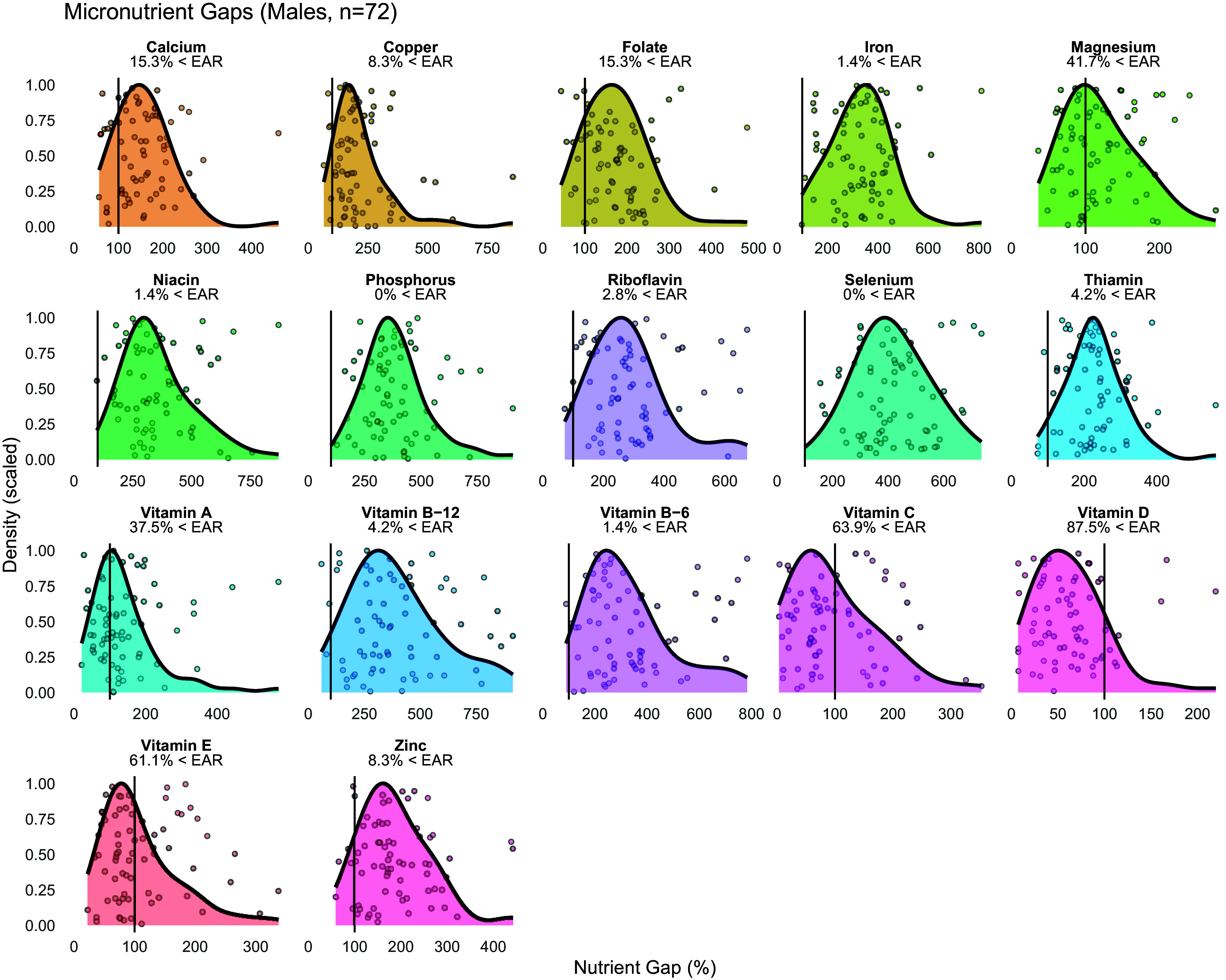
Micronutrient gaps in male participants. Data from seventy-two male participants are displayed. Density plots indicate the distribution of nutrient intakes, expressed as nutrient gap percentages (i.e. percentages of the Estimated Average Requirement [EAR], with a score of 100% indicating intake at the level of the EAR). Vertical black lines in each panel indicate the EAR (i.e. score of 100%) for each nutrient, and the percentage of participants whose intakes were below the EAR is indicated. Values beyond 1000% are not displayed for the purposes of visualization.

In the entire sample, the percentage of participants with vitamin intakes below the EAR ranged from 7.1% for niacin to 91.2% for vitamin D (Figure 1). In female participants, the median nutrient gap score was <100% for vitamin D and vitamin E (*p* ≤ 0.003 for each), did not differ from 100% for folate, vitamin A, or vitamin C after multiplicity correction, and was >100% for the remaining vitamins (Table 3). The percentage of individual female participants with vitamin intakes below the EAR ranged from 9.7% for niacin to 92.9% for vitamin D (Figure 2). In male participants, the median nutrient gap score was <100% for vitamin D (*p* < 0.001; Table 3), did not differ from 100% for vitamin C or vitamin E, and was >100% for the remaining vitamins (Table 3). However, the percentage of individual male participants with vitamin intakes below the EAR ranged from 1.4% for niacin and vitamin B6 to 87.5% for vitamin D (Figure 3). Sex differences were observed for all vitamins, except for vitamin C, indicating higher absolute intakes in male adults as compared to female adults (Table 3). Intakes relative to the EAR were also significantly higher in male participants for all vitamins, except for vitamin C and vitamin A. In the sensitivity analysis, vitamin intakes were slightly higher than in the primary analysis, although overall results were generally similar (Supplementary Table 2; Supplementary Figures 1–3).

**Table 3.** Nutrient intakes and nutrient gaps

		All (*n* = 226)		Female adults (*n* = 154)		Male adults (*n* = 72)		Sex comparison^a^
Nutrient	Intake	Median	IQR	Median	IQR	Median	IQR	*p*
Calcium	Raw (mg)	869.2	652.3	766.3	485.7	1211.4	631.4	<0.001
	Nutrient gap score (%EAR)	108.2	82.9	91.7	57.0	151.4*	78.9	<0.001
Copper	Raw (mcg)	1005.0	688.5	877.3	649.4	1310.6	790.2	<0.001
	Nutrient gap score (%EAR)	143.6	98.4	125.9*	92.6	187.2*	112.9	<0.001
Folate	Raw (mcg)	345.3	302.3	282.3	212.8	547.4	294.3	<0.001
	Nutrient gap score (%EAR)	107.9	94.5	88.0	67.5	171.0*	92.0	<0.001
Iron	Raw (mg)	13.1	10.2	10.4	7.6	20.2	8.5	<0.001
	Nutrient gap score (%EAR)	163.8	160.4	128.8*	93.5	336.7*	142.1	<0.001
Magnesium	Raw (mg)	285.8	200.3	250.7	172.7	363.4	214.2	<0.001
	Nutrient gap score (%EAR)	100.5	67.9	97.0	66.8	110.1	64.9	0.03
Niacin	Raw (mg)	26.3	23.7	19.7	15.8	38.7	20.0	<0.001
	Nutrient gap score (%EAR)	236.4	194.3	179.4*	143.5	322.6*	166.5	<0.001
Phosphorus	Raw (mg)	1456.8	931.5	1230.6	633.7	2073.1	779.0	<0.001
	Nutrient gap score (%EAR)	244.1	169.4	205.8*	114.0	357.4*	135.0	<0.001
Riboflavin	Raw (mg)	2.0	1.6	1.7	1.1	2.9	1.6	<0.001
	Nutrient gap score (%EAR)	207.3	141.7	191.0*	119.3	267.7*	149.0	<0.001
Selenium	Raw (mcg)	117.2	84.8	99.4	57.6	185.7	84.3	<0.001
	Nutrient gap score (%EAR)	260.5	188.4	220.9*	127.9	412.7*	187.4	<0.001
Thiamine	Raw (mg)	1.6	1.2	1.2	0.8	2.2	0.8	<0.001
	Nutrient gap score (%EAR)	165.6	117.1	137.4*	87.7	223.4*	83.3	<0.001
Vitamin A	Raw (mcg RAE)	567.2	521.3	455.3	464.2	722.8	542.1	<0.001
	Nutrient gap score (%EAR)	102.9	93.9	92.3	92.0	115.7*	86.7	0.02
Vitamin B12	Raw (mcg)	4.4	5.4	3.4	3.8	7.2	5.4	<0.001
	Nutrient gap score (%EAR)	219.1	269.0	168.3*	189.0	359.5*	270.9	<0.001
Vitamin B6	Raw (mg)	2.1	2.3	1.7	1.5	3.0	1.8	<0.001
	Nutrient gap score (%EAR)	192.9	210.2	158.7*	133.4	273.8*	168.1	<0.001
Vitamin C	Raw (mg)	50.5	81.7	47.9	76.3	52.1	76.7	0.27
	Nutrient gap score (%EAR)	73.5	119.0	79.8	130.4	69.5	108.1	0.66
Vitamin D	Raw (mcg)	3.4	4.2	2.5	2.9	5.8	4.8	<0.001
	Nutrient gap score (%EAR)	34.0	42.4	25.1*	28.5	58.4*	48.2	<0.001
Vitamin E	Raw (mg)	8.6	8.1	7.6	6.7	10.8	7.8	<0.001
	Nutrient gap score (%EAR)	71.7	67.2	62.9*	55.7	90.3	65.4	<0.001
Zinc	Raw (mg)	10.5	8.0	8.7	5.7	16.2	8.5	<0.001
	Nutrient gap score (%EAR)	142.9	90.4	128.0*	85.4	172.2*	90.5	<0.001

EAR, estimated average requirement; IQR, interquartile range; RAE, retinol activity equivalents.

*Denotes within-sex medians differing from 100% EAR (one-sample Wilcoxon signed-rank tests).

^a^Sex comparisons were performed using Wilcoxon rank-sum tests (i.e. Mann–Whitney *U* tests).

Statistical significance was accepted at *p* ≤ 0.003 (i.e. 0.05/17 nutrients).

### Mineral intakes

In the entire sample, the percentage of participants with mineral intakes below the EAR ranged from 4.0% for phosphorus to 50.0% for magnesium (Figure 1). In female participants, the median nutrient gap score did not differ from 100% for calcium and magnesium (Table 3), with median nutrient gap scores of >100% for copper, iron, phosphorus, selenium, and zinc (*p* < 0.001 for each). The percentage of female participants with mineral intakes below the EAR ranged from 5.8% for phosphorus to 55.8% for calcium (Figure 2). In male participants, the median nutrient gap scores were >100% for all minerals, except for magnesium, which did not differ from 100% (Table 3). However, the percentage of male participants with mineral intakes below the EAR ranged from 0% for phosphorus and selenium to 41.7% for magnesium (Figure 3). Sex differences were observed for all minerals, indicating higher absolute and relative intakes in male participants as compared to female participants, except for the relative intake in magnesium (Table 3). In the sensitivity analysis, mineral intakes were slightly higher than in the primary analysis, although overall results were generally similar (Supplementary Table 2; Supplementary Figures 1–3).

## Discussion

Micronutrient gaps, reflecting lower than recommended intakes of vitamins and minerals, have been demonstrated in the global population, with variation by region and country.^(1,17,18)^ Exercise stresses metabolic pathways that require micronutrients and leads to adaptations that could increase the need for micronutrients.^(12)^ However, there is limited information about the typical vitamin and mineral intakes in exercising adults. The present analyses explored the potential for micronutrient nutrient gaps and sex differences in healthy, exercising adults in the United States. A major finding was that female participants demonstrated lower median intakes than male participants for sixteen of seventeen evaluated vitamins and minerals, as well as lower relative intakes for fourteen of seventeen micronutrients. Median intakes of vitamin D fell below the EAR in both female and male adults, with median intakes of vitamin E below the EAR in female participants only. Importantly, this comparison is conservative as both the median value and EAR are based on group averages. The EAR designates the average daily intake level estimated to meet the requirement of 50% of healthy individuals, meaning that achieving this intake may still be suboptimal in half the population. Additionally, the median within the present sample similarly indicates an average value, such that half of the participants exhibited intakes below this value, even if the median intake is deemed adequate. As such, the percentages of individual participants with intakes falling below the EAR were considered as potentially more informative, as well as being consistent with the presentation of dietary intakes in national surveys, such as *What We Eat in America*.^(8–11)^ Based on this evaluation, micronutrient intakes in female participants fell below the EAR in 6 to 93% of participants in the present study, depending on the specific nutrient being considered, with intakes below the EAR in 0 to 88% of male participants. In the sensitivity analysis, which excluded individuals who potentially misreported energy intake based on a recently described equation developed using doubly-labelled water,^(16)^ nutrient intakes were generally slightly higher, and the percentage of participants consuming less than the EAR was lower (1–90% in female participants and 0–87% in male participants).

National survey data from the United States can be used to compare the intakes observed in the present sample of exercising adults to the broader population.^(8–11)^ For female participants in the current study, the percentage of participants with intakes below the EAR was similar to national data for calcium (55.8% in the present analyses vs. 52–72% in national data), magnesium (53.9% vs. 41–66%), vitamin A (54.5% vs. 37–56%), and zinc (29.9% vs. 18–31%) as indicated by the percent of participants in the present analyses falling within the range of sex-specific percentages across race and ethnicity categories in the national survey data (Table 1). Conversely, the percentage of participants with intakes below the EAR was higher than national data for copper (34.4% vs. 6–24%), folate (57.8% vs. 12–35%), iron (26.6% vs. <3–15%), niacin (9.7% vs. <3–4%), phosphorus (5.8% vs. <3%), riboflavin (12.3% vs. <3–11%), selenium (6.5% vs. <3%), thiamine (22.1% vs. 7–18%), vitamin B12 (27.3% vs. 10–16%), vitamin B6 (25.3% vs. <3–15%), vitamin C (57.8% vs. 25–50%), as indicated by the percentage of participants exceeding sex-specific percentages across all race or ethnicity categories. In contrast, the percentage of participants whose intakes fell below the EAR was slightly lower than national data for vitamin E (77.3% vs. 84–87%) and vitamin D (92.9% vs. >97%). Overall, these results indicate that the micronutrient intakes of active female adults in the present sample were often, but not always, relatively lower than the general population. It has previously been demonstrated that athletes are more likely to display disordered eating as compared to the general population and that eating disorders may be more prevalent in female athletes.^(19–21)^ Low micronutrient intakes have also been reported in female athletes, which may be related to dietary restriction and low energy availability.^(22,23)^ Importantly, the intake of some micronutrients (e.g. vitamin A, folate, and magnesium) may be lower in female athletes as compared to male athletes, with additional differences due to sport classification (i.e. collegiate vs. masters athletes).^(24)^ While speculative, it is possible that the active female adults in the present study exhibited higher degrees of intentional dietary restriction as compared to the general population, as well as male adults, leading to correspondingly lower intakes of micronutrients. Total energy intake in the present analysis was lower in female participants than male participants (average intake of 1748 vs. 2982 kcal/d). While a presentation of micronutrient intakes relative to total energy intake could be performed to examine whether sex differences in micronutrient intakes are present when accounting for total energy intake, we elected to present absolute intakes of micronutrients in the present analyses due to our goal of comparing intakes to the EAR values, which are specified in absolute units and vary by sex. Presenting micronutrient intakes relative to energy intake may obfuscate different absolute micronutrient intakes between sexes, and researchers should consider the specific question being addressed when choosing whether to express absolute or energy-corrected nutrient intakes.

For male participants in the present sample, the percentage of participants with intakes below the EAR was similar to national data for copper (8.3% vs. <3–14%), folate (15.3% vs. 5–18%), iron (1.4% vs. <3–13%), niacin (1.4% vs. <3%), phosphorus (0% vs. <3%), selenium (0% vs. <3%), thiamine (4.2% vs. 3–10%), vitamin B12 (4.2% vs. 4–12%), vitamin B6 (1.4% vs. <3–7%), and as indicated by the percent of participants whose intakes in the present study fell within the range of sex-specific percentages across race and ethnicity categories in the national survey data (Table 1). Conversely, the percentage of participants whose intakes were below the EAR was higher than national data for vitamin C (63.9% vs. 43–58%). However, the percentage of participants with intakes below the EAR was lower than those in national survey data for calcium (15.3% vs. 26–48%), magnesium (41.7% vs. 52–77%), riboflavin (2.8% vs. 4–15%), vitamin D (87.5% vs. 93–96%), vitamin E (61.1% vs. 68–80%), vitamin A (37.5% vs. 48–69%), and zinc (8.3% vs. 17–39%). Collectively, these data indicate that the relationship between the observed micronutrient intakes in exercising male participants and the general population was more heterogeneous than was observed for female participants.

Generally, increased dietary intake or supplementation with vitamins and minerals are recommended only when intakes are inadequate or clinically-defined deficiencies are confirmed.^(12,25)^However, there is debate regarding the physiological accuracy of some clinical biomarkers for assessing nutrient status and identifying deficiencies ^(26–28)^; as such, dietary record and recall data can still be informative when evaluating the contribution of the diet to meeting daily nutritional needs. Nonetheless, it has been noted that, in the absence of correcting deficiency, higher intakes of micronutrients are not typically ergogenic in exercising adults.^(25)^ Conversely, when deficiencies are corrected, some health and exercise performance improvements have been observed.^(29,30)^ While the results of the present investigation do not remove the need for individualized evaluation and clinical testing, as appropriate, they indicate the potential for frequent underconsumption of physiologically important vitamins and minerals in exercising adults. Numerous micronutrients provide support for health and performance in exercising adults, but calcium, vitamin D, iron, and antioxidants (e.g. vitamins A, C, and E) have been noted as micronutrients of particular concern for this population.^(12)^ In the present study, median intakes of vitamin D were low in both sexes, with median intakes of vitamin E also low in female participants. The overlap between nutrients of concern and low observed intakes in the present analyses is noteworthy and provides a rationale for implementing more targeted prospective interventions to evaluate and improve micronutrient status in exercising adults.

As described, the EAR represents a conservative target for recommended nutrient intake since it is the estimated intake level sufficient for half of the population. However, the Dietary Reference Intakes guidelines state that the EAR can be used ‘to examine the probability that usual intake is inadequate’ in individuals, as well as used to ‘estimate the prevalence of inadequate intakes within a group’.^(31)^ The EAR is, by definition, lower than the RDA for any given nutrient.^(15,32)^ Assuming a normal distribution of nutrient requirements among individuals within the population, the RDA is calculated as the EAR plus two standard deviations of the nutrient requirement; therefore, the RDA specifies the intake level that is viewed as sufficient for the vast majority (∼97–98%) of the population. Nevertheless, the Dietary Reference Intakes guidelines explicitly state that the RDA should not be used to assess intakes of groups. Despite this guidance, the guidelines acknowledge that intakes between the EAR and RDA ‘probably need to be improved’, along with statements that intakes below the EAR ‘very likely need to be improved’.^(31)^ Overall, the focus of the present investigation on intakes relative the EAR follows best practices from the Dietary Reference Intake guidelines and is consistent with the presentation of data in national nutritional surveys, despite the somewhat conservative nature of these estimates.

There are several limitations to the present analyses. First, the results are based on exercising adults within one geographical location within the United States. Additionally, participants were predominantly young and associated with one university. As such, the results are not fully generalizable to all exercising adults. While the use of ASA24® allowed for the standardized collection of nutritional intake information, misreporting of intakes may have occurred. Notable underreporting of nutrient intake has been highlighted in recent research using large national datasets,^(16)^ although it is possible underreporting may be of a smaller magnitude in active populations.^(33)^ Although self-reported dietary information has limitations, it still provides potentially meaningful data about estimated nutrient intakes, particularly given the lack of validated biomarkers for several nutrients of interest and debate concerning the suitability of other biomarkers.^(26–28)^ Nonetheless, we performed sensitivity analysis using the method described by Bajunaid et al.,^(16)^ which identified individuals who had potentially misreported energy intake. Although an advantage of this method is the exclusion of potentially implausible energy intakes, the applicability to an active population that may intentionally restrict or augment energy intake beyond the practices of the general population in pursuit of aesthetic and fitness goals has not been specifically explored. As such, we elected to present this analysis as a supplementary sensitivity analysis rather than the primary analysis. An additional limitation of the present work is the possibility that participants did not enter complete dietary supplement information using the ASA24® tool, despite being asked for this information, which could have influenced the apparent nutrient intakes for supplement users. Collectively, despite some limitations, the present analyses contribute to a better understanding of micronutrient intakes in healthy, exercising populations.

In summary, the present analyses of micronutrient intakes in exercising adults revealed widespread sex differences in vitamin and mineral intakes, with female participants reporting lower intakes for nearly all micronutrients. While median intakes below the EAR were relatively uncommon in the present analyses, the intakes of micronutrients were frequently below the EAR in meaningful percentages of individuals, indicating the need for increased consumption in portions of the active population. Collectively, the present analyses indicate varying degrees of underconsumption of micronutrients, particularly in exercising female adults. The potential to improve vitamin and mineral intakes, micronutrient status, and attendant health and performance outcomes through targeted dietary and supplementation interventions in exercising adults should be explored in future research.

## Supporting information

Tinsley et al. supplementary material 1 Tinsley et al. supplementary material

Tinsley et al. supplementary material 2 Tinsley et al. supplementary material

Tinsley et al. supplementary material 3 Tinsley et al. supplementary material

Tinsley et al. supplementary material 4 Tinsley et al. supplementary material

Tinsley et al. supplementary material 5 Tinsley et al. supplementary material

## Data Availability

Data and analysis code may be available upon request, pending necessary institutional approvals.

## Supplementary material

The supplementary material for this article can be found at https://doi.org/10.1017/jns.2025.10070.
