# Supplementary figures and images for: Sex differences in nutrient gaps among active adults
Source: J Nutr Sci. 2026 Jan 2;15:e5. doi: 10.1017/jns.2025.10070 (PMC12800541; doi:10.1017/jns.2025.10070)

# Micronutrient Gaps (n=159) – Sensitivity Analysis

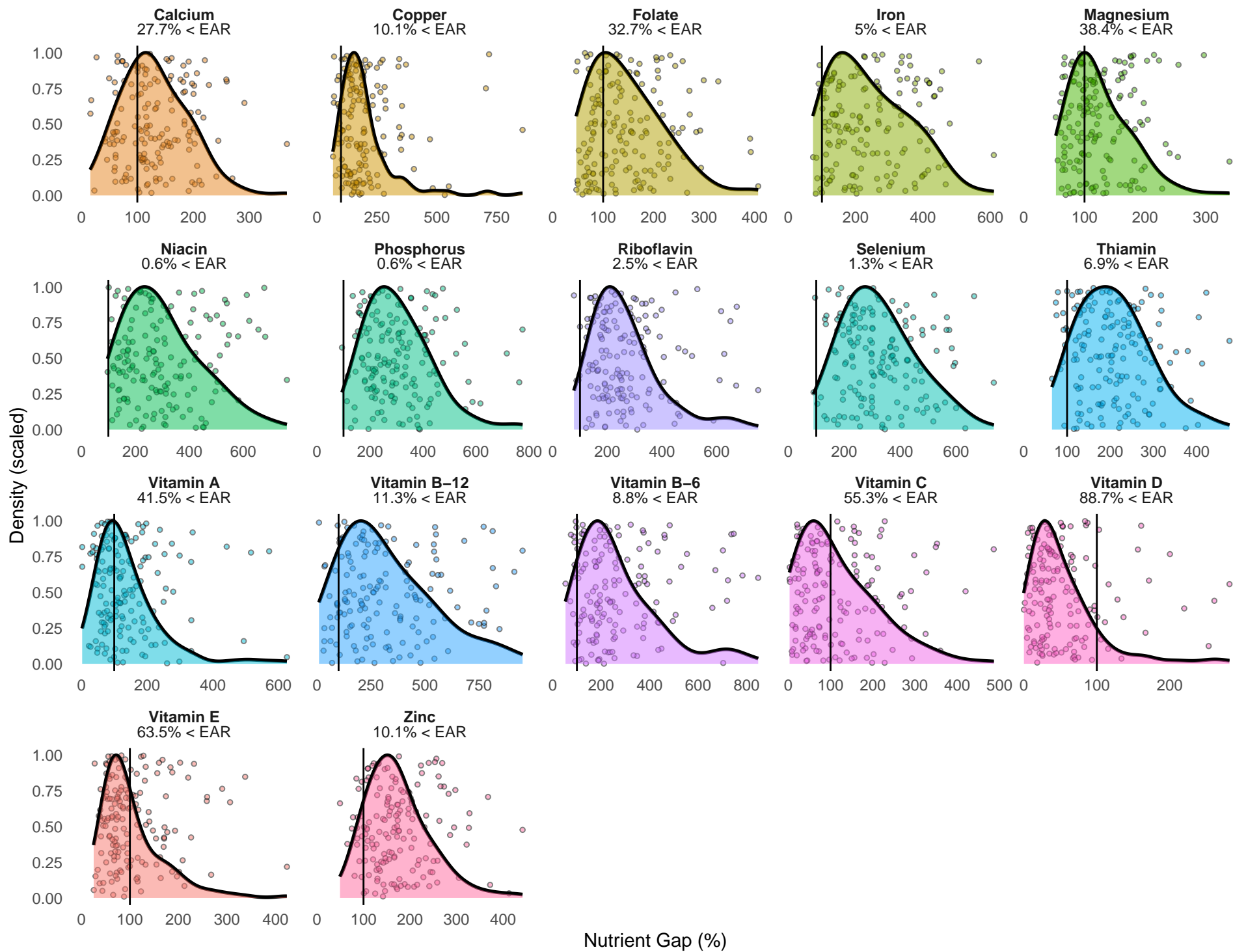

Supplement: Tinsley et al. supplementary material 1 — Tinsley et al. supplementary material [file S2048679025100700sup001.pdf]

# Micronutrient Gaps (Females, n=98) – Sensitivity Analysis

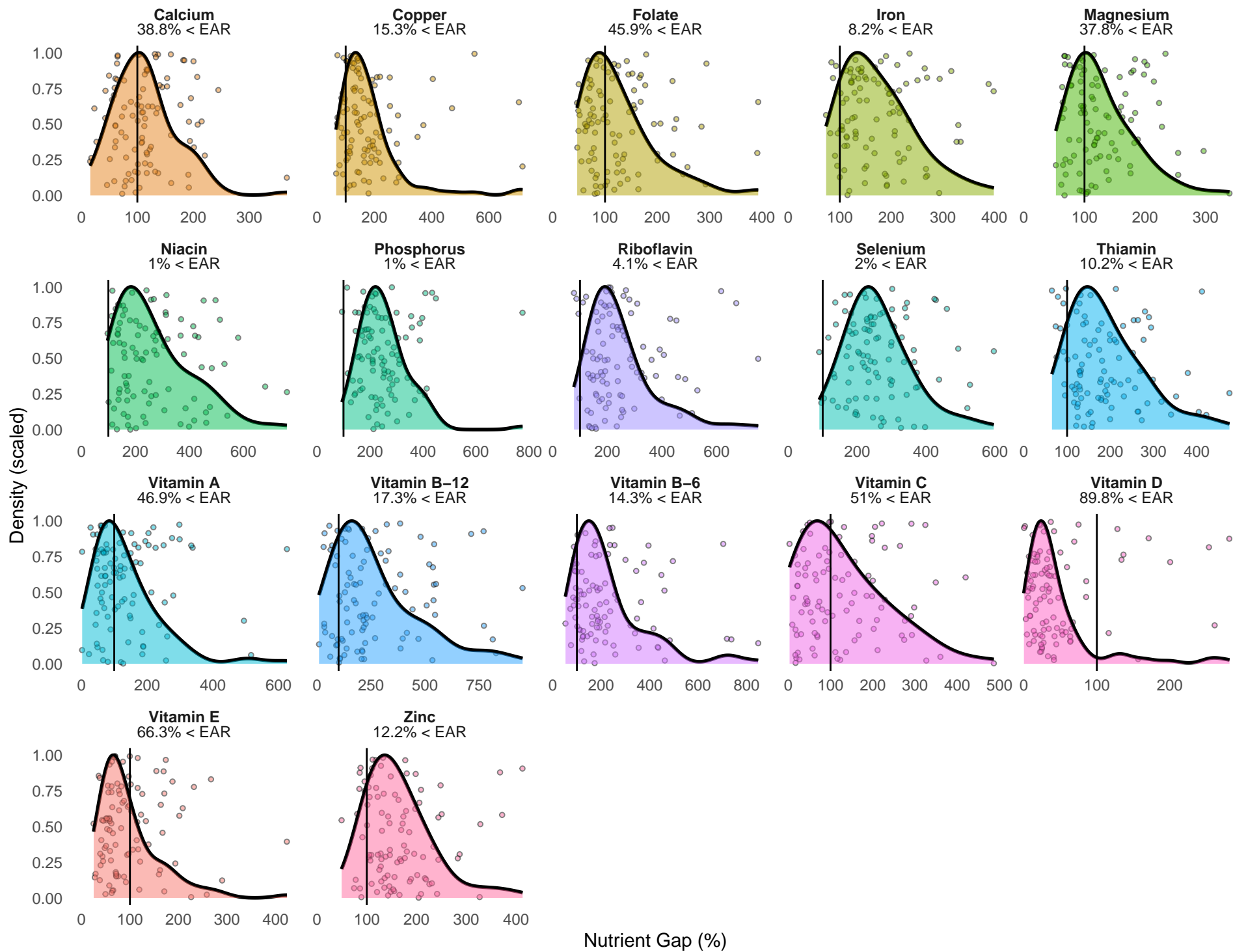

Supplement: Tinsley et al. supplementary material 2 — Tinsley et al. supplementary material [file S2048679025100700sup002.pdf]

# Micronutrient Gaps (Males, n=61) – Sensitivity Analysis

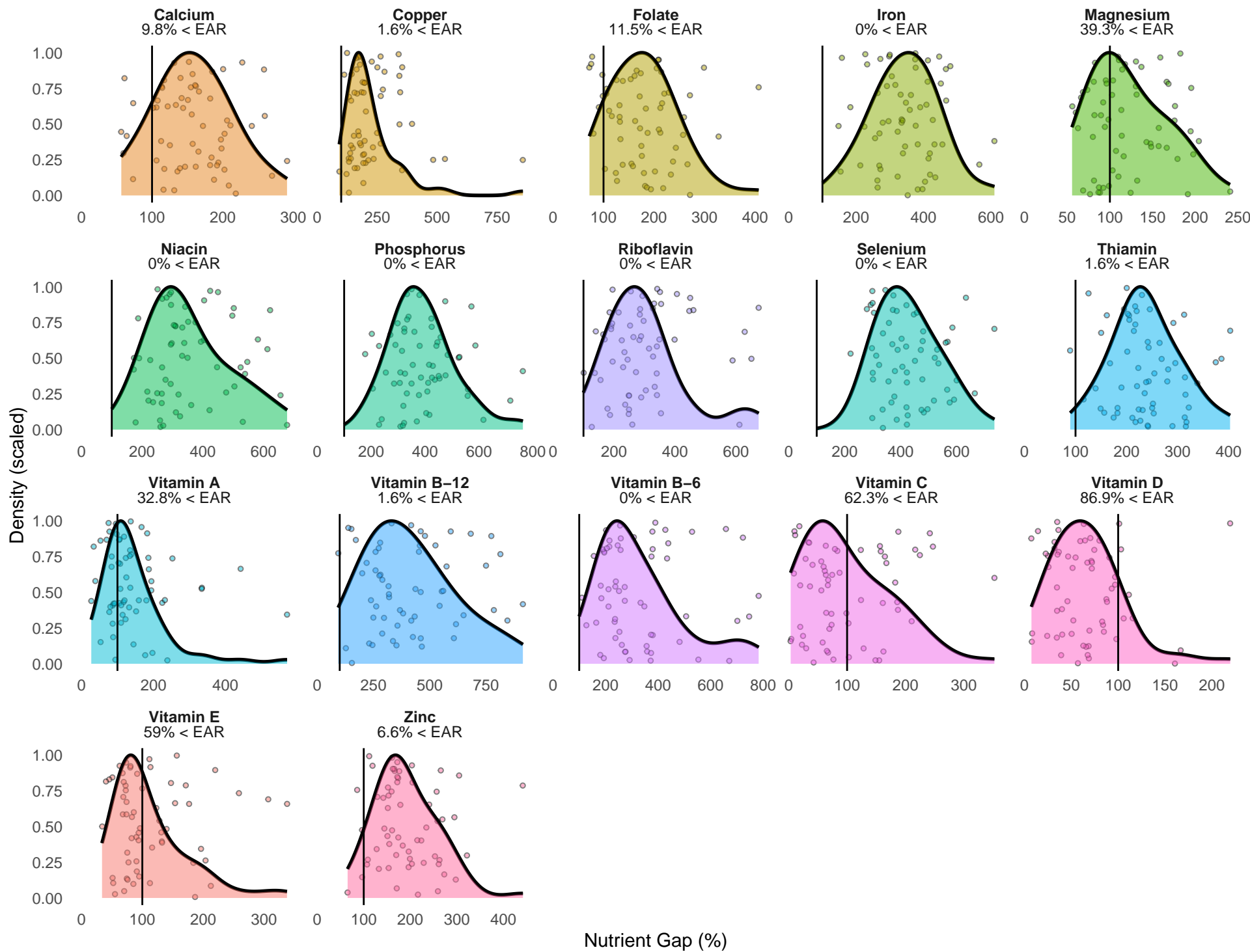

Supplement: Tinsley et al. supplementary material 3 — Tinsley et al. supplementary material [file S2048679025100700sup003.pdf]
