# Supplementary material for: Sex differences in nutrient gaps among active adults
Source: J Nutr Sci. 2026 Jan 2;15:e5. doi: 10.1017/jns.2025.10070 (PMC12800541; doi:10.1017/jns.2025.10070)
Supplement: Tinsley et al. supplementary material 4 — Tinsley et al. supplementary material [file S2048679025100700sup004.docx]

**Supplementary Table 1. Participant Characteristics (Sensitivity Analysis)**

|  | **All (n=159)** | | **F (n=98)** | | **M (n=61)** | |
| --- | --- | --- | --- | --- | --- | --- |
| **Variable** | **Mean** | **SD** | **Mean** | **SD** | **Mean** | **SD** |
| Age (y) | 22.8 | 3.8 | 22.7 | 3.7 | 23.0 | 4.0 |
| Height (cm) | 168.1 | 9.2 | 163.1 | 6.5 | 176.2 | 6.7 |
| Body Mass (kg) | 70.1 | 12.8 | 64.6 | 10.1 | 79.0 | 11.6 |
| BMI (kg/m^2^) | 24.7 | 3.4 | 24.2 | 3.3 | 25.5 | 3.5 |
| Body Fat (%) | 26.3 | 8.5 | 31.2 | 6.3 | 18.6 | 5.0 |
| FFMI (kg/m^2^) | 18.1 | 3.0 | 16.5 | 1.8 | 20.7 | 2.5 |
| Energy Intake (kcal/d) | 2419 | 774 | 2036 | 494 | 3033 | 750 |
| Years Trained^1^ | 6.1 | 4.8 | 5.8 | 4.8 | 7.3 | 4.4 |
| Exercise Frequency (d/week)^1^ | 3.8 | 1.6 | 3.5 | 1.6 | 4.8 | 1.3 |
| RT Frequency (d/week)^1^ | 2.4 | 1.9 | 2.0 | 1.8 | 3.9 | 1.6 |
| ET Frequency (d/week)^1^ | 1.7 | 1.4 | 1.7 | 1.4 | 1.5 | 1.2 |

^1^Sample sizes for exercise history questionnaire were n=102 (all), n=83 (F), and n=19 (M).

*Abbreviations:* M (male), F (female), SD (standard deviation), BMI (body mass index), FFMI (fat-free mass index), RT (resistance training), ET (endurance training)
