# Supplementary material for: Sex differences in nutrient gaps among active adults
Source: J Nutr Sci. 2026 Jan 2;15:e5. doi: 10.1017/jns.2025.10070 (PMC12800541; doi:10.1017/jns.2025.10070)
Supplement: Tinsley et al. supplementary material 5 — Tinsley et al. supplementary material [file S2048679025100700sup005.docx]

**Supplementary Table 2. Nutrient Intakes and Nutrient Gaps (Sensitivity Analysis)**

|  |  | **All**  **(n=159)** | | **Female Adults**  **(n=98)** | | **Male Adults**  **(n=61)** | | **Sex Comparison^1^** |
| --- | --- | --- | --- | --- | --- | --- | --- | --- |
| **Nutrient** | **Intake** | **Median** | **IQR** | **Median** | **IQR** | **Median** | **IQR** | ***p*** |
| Calcium | Raw (mg) | 979.7 | 607.3 | 869.2 | 509.2 | 1250.6 | 612.7 | <0.001 |
|  | Nutrient Gap Score (%EAR) | 119.2 | 75.9 | 108.4 | 63.0 | 156.3* | 76.6 | <0.001 |
| Copper | Raw (mcg) | 1167.3 | 583.1 | 1033.2 | 599.6 | 1332.2 | 669.6 | <0.001 |
|  | Nutrient Gap Score (%EAR) | 166.8 | 82.8 | 149.2* | 85.7 | 190.3* | 95.7 | <0.001 |
| Folate | Raw (mcg) | 421.4 | 325.0 | 334.0 | 252.6 | 559.9 | 264.9 | <0.001 |
|  | Nutrient Gap Score (%EAR) | 131.7 | 101.6 | 104.4 | 78.9 | 175.0* | 82.8 | <0.001 |
| Iron | Raw (mg) | 16.1 | 10.6 | 12.2 | 7.5 | 20.7 | 8.4 | <0.001 |
|  | Nutrient Gap Score (%EAR) | 213.8 | 189.8 | 151.6* | 92.2 | 345.6* | 140.0 | <0.001 |
| Magnesium | Raw (mg) | 314.8 | 188.8 | 295.4 | 166.4 | 364.2 | 226.2 | <0.001 |
|  | Nutrient Gap Score (%EAR) | 113.7 | 65.4 | 114.9* | 65.6 | 110.4* | 68.6 | 0.72 |
| Niacin | Raw (mg) | 31.7 | 24.5 | 25.5 | 21.7 | 38.4 | 21.8 | <0.001 |
|  | Nutrient Gap Score (%EAR) | 275.1 | 212.1 | 231.9* | 197.6 | 319.9* | 181.2 | <0.001 |
| Phosphorus | Raw (mg) | 1679.6 | 920.0 | 1420.9 | 552.1 | 2188.7 | 733.2 | <0.001 |
|  | Nutrient Gap Score (%EAR) | 284.5 | 166.1 | 235.8* | 99.0 | 377.4* | 128.1 | <0.001 |
| Riboflavin | Raw (mg) | 2.3 | 1.6 | 1.9 | 1.1 | 3.1 | 1.4 | <0.001 |
|  | Nutrient Gap Score (%EAR) | 239.7 | 143.2 | 208.5* | 122.7 | 283.4* | 126.1 | 0.001 |
| Selenium | Raw (mcg) | 135.6 | 82.7 | 109.7 | 48.5 | 189.2 | 67.8 | <0.001 |
|  | Nutrient Gap Score (%EAR) | 301.4 | 183.9 | 243.7* | 107.7 | 420.5* | 150.6 | <0.001 |
| Thiamin | Raw (mg) | 1.9 | 1.2 | 1.6 | 0.9 | 2.3 | 0.8 | <0.001 |
|  | Nutrient Gap Score (%EAR) | 201.2 | 113.7 | 173.3* | 102.4 | 231.5* | 78.9 | <0.001 |
| Vitamin A | Raw (mcg RAE) | 635.3 | 561.6 | 536.0 | 561.3 | 739.6 | 547.5 | 0.001 |
|  | Nutrient Gap Score (%EAR) | 116.6 | 104.3 | 108.4 | 112.3 | 118.3* | 87.6 | 0.019 |
| Vitamin B12 | Raw (mcg) | 5.3 | 6.1 | 4.2 | 4.7 | 7.6 | 5.6 | <0.001 |
|  | Nutrient Gap Score (%EAR) | 265.9 | 304.8 | 208.8* | 232.7 | 381.9* | 279.6 | <0.001 |
| Vitamin B6 | Raw (mg) | 2.4 | 2.2 | 2.0 | 1.5 | 3.0 | 1.8 | <0.001 |
|  | Nutrient Gap Score (%EAR) | 217.9 | 199.2 | 181.9* | 138.0 | 273.3* | 165.6 | <0.001 |
| Vitamin C | Raw (mg) | 56.0 | 90.5 | 58.4 | 91.8 | 54.7 | 85.9 | 0.91 |
|  | Nutrient Gap Score (%EAR) | 87.2 | 123.2 | 97.4 | 153.0 | 73.0 | 115.7 | 0.13 |
| Vitamin D | Raw (mcg) | 3.7 | 4.6 | 3.0 | 3.4 | 6.4 | 4.8 | <0.001 |
|  | Nutrient Gap Score (%EAR) | 36.9 | 46.2 | 29.8* | 33.5 | 64.4* | 48.1 | <0.001 |
| Vitamin E | Raw (mg) | 9.9 | 7.7 | 9.2 | 7.3 | 10.9 | 7.1 | 0.03 |
|  | Nutrient Gap Score (%EAR) | 82.8 | 64.2 | 76.6 | 60.9 | 90.8 | 59.2 | <0.001 |
| Zinc | Raw (mg) | 12.5 | 7.4 | 10.1 | 5.4 | 16.4 | 7.8 | <0.001 |
|  | Nutrient Gap Score (%EAR) | 164.0 | 79.1 | 147.5* | 78.4 | 174.0* | 82.7 | 0.003 |

* denotes within-sex medians differing from 100% EAR (one-sample Wilcoxon signed-rank tests).

^1^Sex comparisons were performed using Wilcoxon rank-sum tests (i.e., Mann-Whitney U tests).

Statistical significance was accepted at *p*≤0.003 (i.e., 0.05 / 17 nutrients).

*Abbreviations:* mg (milligram), mcg (microgram), EAR (estimated average requirement), IQR (interquartile range), RAE (retinol activity equivalents)
